# Research Progress on Bidirectional Regulation of the Microbiota–Gut–Brain Axis in Autism Spectrum Disorder Based on the Immune–Metabolic–Endocrine Interactive Network

**DOI:** 10.3390/biom16091321

**Published:** 2026-09-11

**Authors:** Weiao Kong, Haoke Qiu, Yuhang Jiang, Huanhuan Ge, Wanyi Wu, Lefan Huang, Lisheng Chu, Lijun Ge

**Affiliations:** 1 College of Life Science, Zhejiang Chinese Medical University, Hangzhou 310053, China; 202411115311005@zcmu.edu.cn (W.K.); 202511115311006@zcmu.edu.cn (H.Q.); 202611113811003@zcmu.edu.cn (Y.J.); 20215060005@zcmu.edu.cn (H.G.); 202212213903013@zcmu.edu.cn (W.W.); 202312213903015@zcmu.edu.cn (L.H.); 2 Key Laboratory of TCM Encephalopathy of Zhejiang Province, Zhejiang Chinese Medical University, Hangzhou 310053, China; 3 Department of Physiology, Zhejiang Chinese Medical University, Hangzhou 310053, China

**Keywords:** autism spectrum disorder, microbiota–gut–brain axis, immune–metabolic–endocrine interaction, gut microbiota dysbiosis, neuroinflammation, targeted intervention

## Abstract

Autism spectrum disorder (ASD) is a highly heterogeneous neurodevelopmental disorder characterized by core features of social communication deficits and high prevalence of gastrointestinal comorbidities. With its continuously rising global prevalence, current therapeutic modalities remain unable to target and ameliorate the core symptoms of ASD. The microbiota–gut–brain axis (MGBA), a critical pathway mediating crosstalk between the gut microbiota and the brain, has been extensively documented to be deeply involved in the pathological progression of ASD in recent years. However, prior studies have predominantly focused on the unidirectional regulation of the brain by gut microbiota, lacking an integrated account of the bidirectional regulation across immune, metabolic, and endocrine systems. Centered on the immune–metabolic–endocrine interactive network, this review systematically delineates the bidirectional regulatory mechanisms of the MGBA in ASD by integrating recent evidence from microbiota sequencing, animal models, and clinical intervention studies, with the aim of clarifying the bidirectional causal controversy between intestinal microecological disturbance and ASD behavioral abnormalities. This review proposes that in children with ASD, decreased abundance of beneficial intestinal bacteria and disrupted metabolic profiles of short-chain fatty acids synergistically impair intestinal barrier integrity, triggering peripheral chronic inflammation that further drives excessive microglial activation-mediated central neuroinflammation. Subsequently, disturbances in the homeostasis of multiple neurotransmitters including 5-hydroxytryptamine (5-HT), γ-aminobutyric acid (GABA), histamine, and dopamine occur via the vagus nerve and hypothalamic–pituitary–adrenal (HPA) axis, ultimately driving ASD behavioral abnormalities. Conversely, chronic stress and behavioral characteristics associated with ASD reshape the intestinal microecology through neuroendocrine pathways, forming a vicious cycle of “microbiota dysbiosis—immune inflammation—HPA axis hyperactivity—further intestinal microecological imbalance”. This review summarizes the therapeutic efficacy and translational bottlenecks of three types of microecological interventions: fecal microbiota transplantation (FMT), probiotics, and ketogenic diet, and analyzes the current limitations in the field, including pronounced population heterogeneity, unclear cross-talk mechanisms among multiple pathways, and the scarcity of large-sample clinical evidence. Collectively, this review preliminarily elucidates the complete multi-system interactive framework of MGBA regulation in ASD, providing theoretical support for mechanistic research and gut-targeted individualized interventions for ASD.

## 1. Introduction

Autism spectrum disorder (ASD) is a neurodevelopmental disorder characterized by core features of social communication deficits, repetitive stereotyped behaviors, and restricted interests [1]. According to the latest 2025 data from the U.S. Centers for Disease Control and Prevention (CDC), the prevalence of ASD among children in the United States has risen to 3.2%, representing a fivefold increase since surveillance began in 2000 [2]. Over the past decade, the prevalence of ASD among children in China has shown a continuous upward trend. Statistics indicate that the pooled prevalence from 2017 to 2023 is significantly higher than that in earlier years; the total number of individuals with ASD spectrum disorders nationwide has exceeded 14 million, including approximately 2 million children aged 0–14 years, with around 160,000 newly diagnosed cases each year [3]. ASD has become a prominent and pressing public health issue for children in China. Most recently, in July 2026, the State Council Working Committee on Persons with Disabilities included autism as a dedicated section in the five-year plan for the first time [4]. The high prevalence of ASD not only imposes severe psychological and caregiving burdens on patients’ families but also generates substantial socioeconomic costs. In the United States, for instance, the annual medical and care costs associated with ASD reached $223 billion nationwide in 2020 [5]. However, as the pathogenesis of ASD has not been fully elucidated, there remains a lack of effective clinical treatments targeting its core symptoms, posing great challenges to the prevention and control of this disorder.

In recent years, the critical role of microbiota–gut–brain axis (MGBA) dysfunction in the pathogenesis of ASD has received growing attention. Numerous clinical observations have shown that patients with ASD are frequently accompanied by severe gastrointestinal symptoms, including constipation, abdominal pain, bloating, and diarrhea. For example, a survey by Frye et al. revealed that approximately 91% of children with ASD experience varying degrees of gastrointestinal dysfunction [6,7]. This high comorbidity rate strongly suggests a close association between gastrointestinal impairment and MGBA dysfunction in ASD. Currently, MGBA dysfunction is regarded as a critical pathway linking intestinal abnormalities to neurobehavioral phenotypes. Nevertheless, a key scientific controversy remains in the field: is gut microbiota dysbiosis a pathogenic trigger of ASD, or a secondary alteration resulting from the pathological state of ASD? Taniya et al. [8] proposed that multiple factors such as unhealthy diet and microbial infection can induce maternal microbiota dysbiosis, which in turn disrupts neurodevelopment in offspring and leads to lifelong behavioral deficits. Conversely, Wang et al. [9] compared fecal samples from children with ASD and typically developing children using metagenomic sequencing, and identified aberrant microbial epitopes (MEs) in the gut of children with ASD. They further observed that alterations in these MEs were correlated with changes in gut microbiota structure and abnormal intestinal IgA levels, suggesting that intestinal microecological imbalance in ASD may trigger local immune perturbation and barrier damage by generating abnormal antigenic epitopes. Although the causal relationship between the two has not been fully clarified, the interaction between intestinal microecology and phenotypes is most likely mediated through the bidirectional regulatory network of the MGBA.

**Figure 1:**
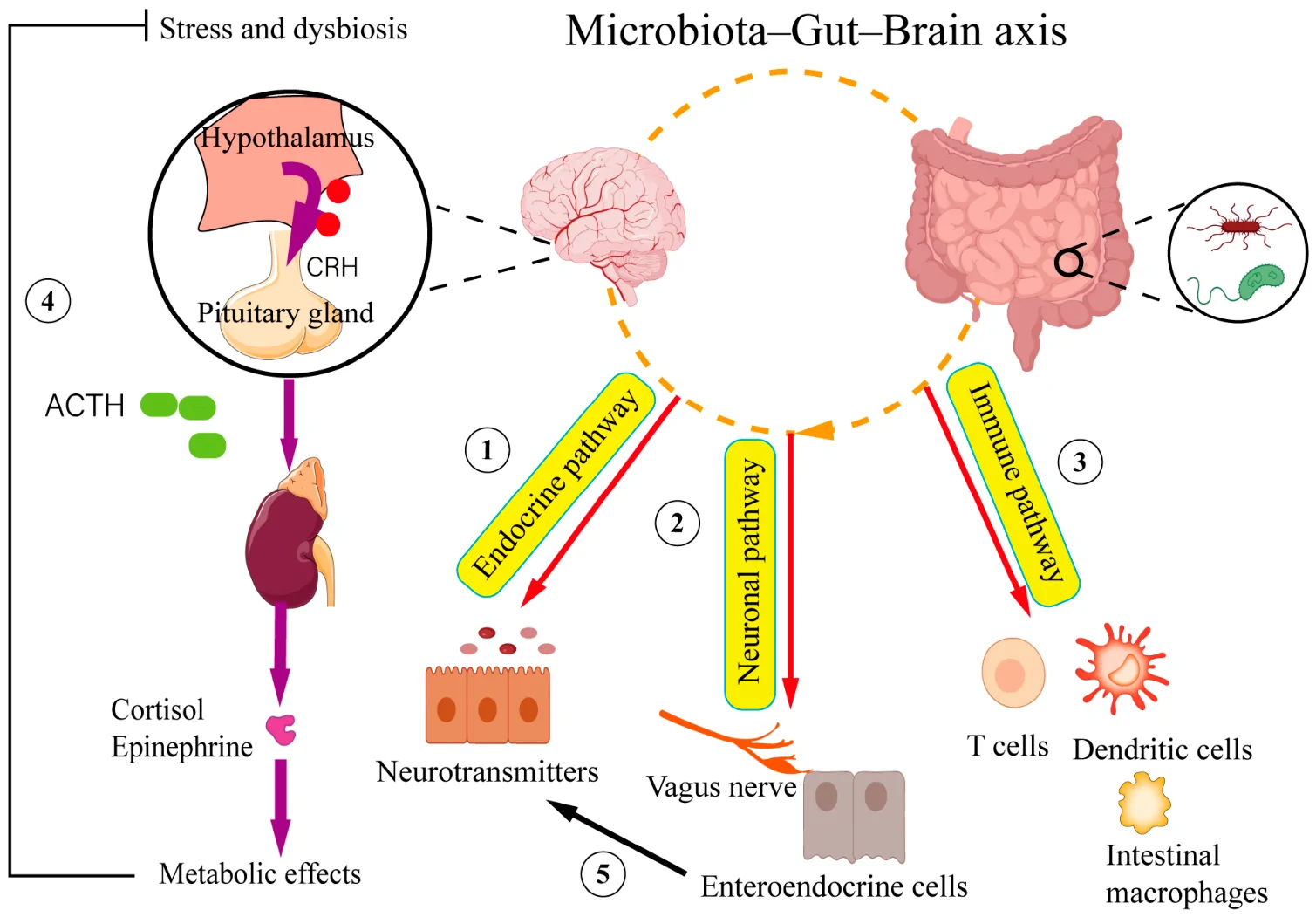
The bidirectional communication of the MGBA is mediated via the HPA axis, immune, endocrine, and neural pathways. **Notes:** ① Endocrine pathway: mediates signal communication between the brain and the gut via neurotransmitters. ② Neural pathway: the vagus nerve connects enteroendocrine cells and mediates neural signal transmission between the brain and the intestine. ③ Immune pathway: gut microbiota regulates immune cell function and participates in the immune modulation of the gut–brain axis. ④ HPA axis: hormones are generated through cascade reactions to induce metabolic effects, while stress and microbiota dysbiosis act back on the HPA axis to form a regulatory loop. ⑤ Enteroendocrine cells: synthesize and secrete neurotransmitters, which serve as core signaling molecules of the endocrine pathway.

The MGBA is a bidirectional signaling network formed by the synergy of the central nervous system, enteric nervous system, autonomic nervous system, and immune–endocrine system. It mediates gut–brain crosstalk via multiple pathways, including metabolites, neurotransmitters, immune inflammation, and the hypothalamic–pituitary–adrenal (HPA) axis [10]. The brain can regulate intestinal barrier function, microbiota structure, and metabolic function via descending pathways; in turn, gut microorganisms modulate neuroinflammation and brain function through bioactive molecules, thereby affecting behavioral phenotypes (Figure 1). Given the pivotal role of the immune–metabolic–endocrine system in the MGBA, this review systematically summarizes the mechanistic advances of bidirectional MGBA regulation in ASD, with a focus on integrating multi-system interactive network pathways and exploring its potential as an intervention target. This work aims to provide theoretical basis and novel insights for elucidating the pathogenesis of ASD and developing microecology-targeted prevention and treatment strategies.

## 2. Gut Microbiota Dysbiosis and Bidirectional Causality Controversy in ASD Within the MGBA Regulatory Network

The gut microbiota serves as the core functional component of the MGBA. Its community structure and metabolic homeostasis participate in host neurodevelopment, immune tolerance, and behavioral regulation through the immune–metabolic–endocrine interactive network. To date, multiple studies based on 16S rRNA gene sequencing and shotgun metagenomic sequencing have confirmed significant differences in the composition, diversity, and functional profiles of gut microbiota between children with ASD and healthy controls (Table 1), providing epidemiological evidence for the involvement of the MGBA in the pathological progression of ASD. Nevertheless, the causal direction, temporal sequence, and mediating pathways between gut microbiota dysbiosis and ASD remain core controversies in the field. Existing evidence more strongly supports a dynamic regulatory pattern of bidirectional interaction and mutual reinforcement.

### 2.1. The Dysbiosis-Initiation Hypothesis

**Figure 2:**
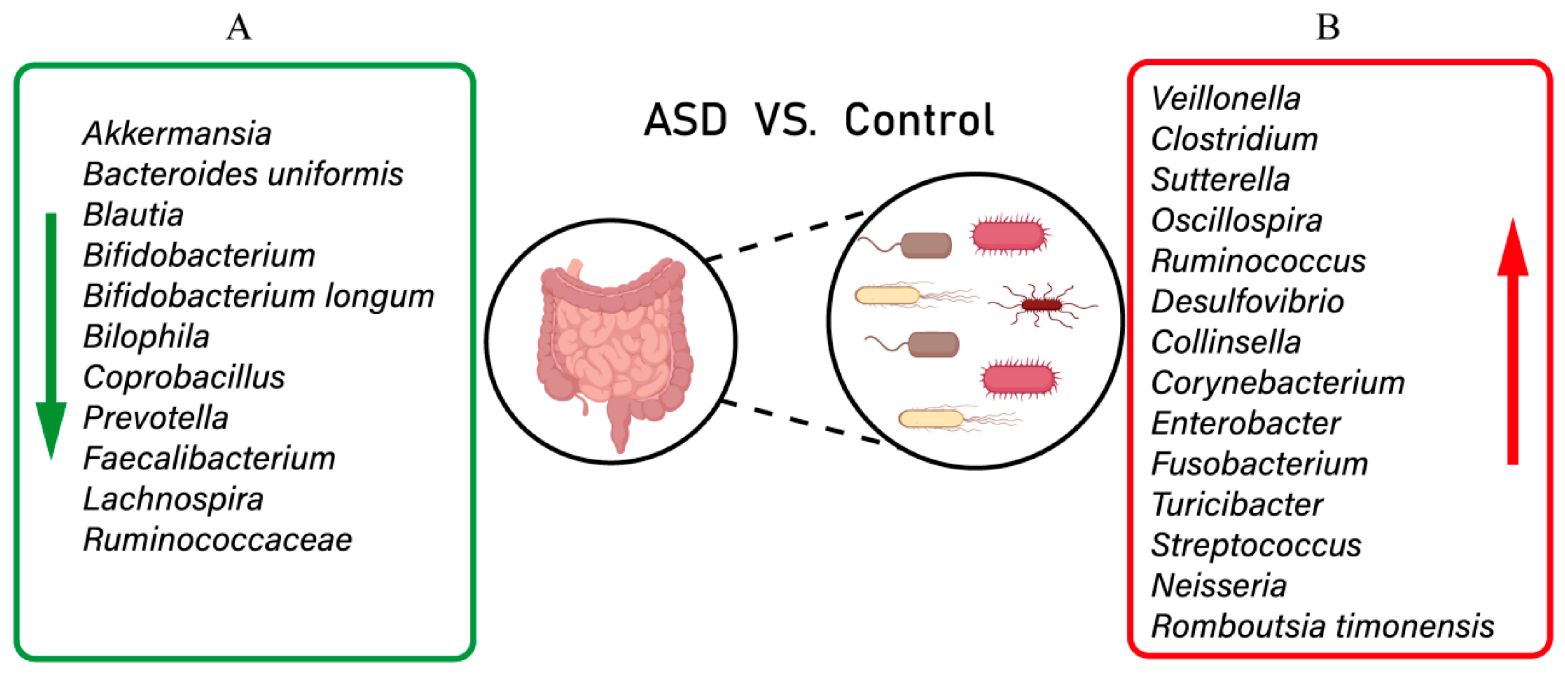
Commonly altered bacterial taxa in the gut of patients with ASD. **Notes:** A: Decreased abundance of beneficial bacterial flora in the gut of patients with ASD; B: increased abundance of potentially pathogenic bacterial flora in the gut of patients with ASD. This schematic diagram is summarized from multiple gut-microbiome studies of ASD cohorts [22,23,24].

A subset of studies supports the dysbiosis-initiation hypothesis, which posits that gut microbiota dysbiosis acts as an upstream initiating factor driving the pathological progression of ASD. For instance, multiple 16S rRNA sequencing studies have revealed that compared with healthy controls, children with ASD exhibit significantly reduced abundance of beneficial commensal bacteria such as *Barnesiella*, *Prevotella*, and *Bifidobacterium*, alongside an over-enrichment of opportunistic pathogens including *Megamonas*, *Ruminococcus*, *Fusobacterium*, and *Desulfovibrio* [22,23,24] (Figure 2). These structural alterations in the microbiota are strongly correlated with common gastrointestinal symptoms in patients with ASD, such as constipation and diarrhea, and directly disrupt metabolic and immune homeostasis. Li et al. [14] found an elevated *Firmicutes*/*Bacteroidetes* (F/B) ratio in the gut microbiota of children with ASD, primarily driven by decreased abundance of Bacteroidetes. This feature accounts for the widespread polysaccharide metabolism disorders observed in children with ASD from a metabolic functional perspective. Alterations in gut microbiota can further induce changes in the immune system. Dan et al. [11] demonstrated a reduced abundance of *Prevotella*, a genus capable of degrading polysaccharides to produce succinate, in the gut of patients with ASD. Previous studies have confirmed that *Prevotella*-mediated succinate signaling enhances T-cell immune responses, and its decreased abundance may contribute to immune regulatory dysfunction in patients with ASD [25]. Sharon et al. [26] colonized germ-free mice with gut microbiota from children with ASD via fecal microbiota transplantation (FMT), and observed ASD-like behavioral phenotypes in the recipient mice. Furthermore, prospective birth-cohort studies further support that microbial perturbations can emerge before the onset of clinical ASD symptoms. Ahrens et al. [27] identified characteristic gut microbiota differences in infants who were later diagnosed with ASD, even before typical autistic behavioral manifestations appeared. Decreased linolenic acid levels and elevated per-and polyfluoroalkyl substance concentrations were also detected in neonatal umbilical cord blood. These substances may interfere with early-life neurodevelopment and participate in the regulation of disease susceptibility to ASD, providing direct experimental evidence that gut microbiota dysbiosis mediates behavioral abnormalities.

### 2.2. The Reverse Regulation Hypothesis

Other studies have proposed the reverse regulation hypothesis, which holds that neurodevelopmental abnormalities and behavioral characteristics of ASD reshape the intestinal microecology in reverse, resulting in behavior-driven microbiota dysbiosis. Studies supporting this hypothesis mainly focus on the impacts of the core behavioral and physiological features of ASD on the intestinal microenvironment. For example, Yap et al. [28] found that the typical restrictive dietary preferences of children with ASD are significantly associated with reduced gut microbiota diversity. It is speculated that insufficient dietary fiber intake and altered intestinal nutritional substrates caused by selective eating may indirectly lead to decreased abundance of beneficial commensal bacteria such as *Bifidobacterium*. Furthermore, Hosie et al. [29] observed in a mouse model carrying ASD-related symptomatic mutations that abnormal synaptic function of the enteric nervous system (ENS) precedes gut microbiota dysbiosis, accompanied by gastrointestinal motility dysfunction. Clinical cross-sectional studies have revealed that the frequency of stereotyped behaviors in children with ASD is positively correlated with the severity of gut microbiota dysbiosis, and the microbiota structure of some children shows a certain degree of improvement after behavioral intervention [21], providing correlational evidence for the reverse regulation of microbiota by behavior. Evidence from genetically defined neurodevelopmental disease models further supports such brain-to-gut signaling within MGBA. For example, Wang and colleagues detected alterations in gut microbiota and metabolome in female heterozygous Mecp2-e1 mice prior to the onset of prominent neuromotor symptoms. Intestinal immune disturbance, by contrast, mostly manifested at the late stage of disease. These findings suggest that gut microbiota and metabolic perturbations can amplify disease progression of Rett syndrome, yet they do not serve as the initiating trigger for this disorder. This study also highlights marked sexual dimorphism in Rett syndrome pathology, as intestinal molecular phenotypes observed in male hemizygous Mecp2-e1 mice show poor consistency with those of human Rett patients [30]. In addition, a human cross-sectional cohort study conducted by Strati F and co-workers identified subclinical intestinal inflammation indicated by elevated fecal calprotectin among Rett patients. These patients presented concurrent dysbiosis of both bacterial and fungal communities in the gut, and such microbial imbalance occurred independent of constipation status. Dysregulated gut microbiota generates aberrant short-chain fatty acid profiles, which further contribute to gastrointestinal pathological processes in Rett syndrome [31]. These observations reinforce the concept that primary neurodevelopmental defects can drive secondary gut ecological disruption, a key mechanism underlying brain-to-gut signaling within MGBA.

### 2.3. Heterogeneity and Limitations of Current Studies

Notably, the results of existing studies exhibit heterogeneity, with inconsistent trends in the abundance of certain bacterial taxa across different cohorts, which hinders the formation of mechanistic consensus. For example, Abuljadayel et al. [32] found an increased relative abundance of *Lactobacillus* in the gut of patients with ASD. Conversely, He et al. [33] reported that the abundance of *Lactobacillus* in the gut of children with ASD was significantly lower than that in typically developing children. Such inconsistencies in research findings may be attributed to multiple factors, including geographic distribution, age structure, dietary habits, disease severity, and differences in sequencing techniques across study samples.

Furthermore, current studies predominantly focus on bacterial communities, with relatively insufficient attention to fungal components. Existing studies have shown increased abundance of *Candida* in the gut of children with ASD, and the mycotoxins produced by it may induce neuroinflammation and exacerbate the pathological state of ASD [34]. Francesco et al. [35] similarly indicated that overgrowth of *Candida* participates in the pathogenesis of ASD by altering the overall structure of the microbial community. However, the study by Chamtouri et al. [36] found no significant correlation between *Candida* abundance and ASD. Accordingly, current findings regarding gut fungi remain highly inconsistent. Alookaran et al. [37] performed ITS sequencing to analyze fecal mycobiome profiles among 50 children with ASD with or without gastrointestinal symptoms and typically developing controls. No differences in fungal alpha-diversity were detected across the three groups. Notably, detection rates of *Candida* showed no statistical difference among groups, and the presence of *Candida* was not correlated with either behavioral or gastrointestinal symptoms. In addition, fecal calprotectin and serum dectin-1 levels were also unaltered, indicating no abnormal activation of fungal-directed immunity in children with ASD. These conflicting results suggest that evidence supporting a causal link between gut fungi and ASD pathogenesis remains weak. Some studies have detected increased abundance of *Saccharomyces cerevisiae* in a subset of patients with ASD, and speculated that it may influence disease progression through immune regulatory mechanisms [38]. Overall, research on the role of gut fungi in ASD remains relatively limited, and the specific regulatory mechanisms have not yet been clarified, representing an important direction for future research.

Taken together, there is no unidirectional causal relationship between microbiota dysbiosis and ASD. Current evidence supports that the two form a bidirectional interactive regulatory association via the MGBA. Microbiota abnormalities may act as an initiating factor to affect neurodevelopment through immune, metabolic, and endocrine pathways, while ASD-related behaviors, stress, and gastrointestinal dysfunction may in turn reshape the intestinal microecology. Currently, research in this field still faces challenges including the high heterogeneity of results, insufficient research on microbial components such as fungi, causal evidence relying heavily on animal models, and unclear mediating pathways. The following sections systematically analyze the pathological cascade processes and molecular mechanisms underlying the bidirectional regulation of ASD by the MGBA, focusing on the immune–metabolic–endocrine interactive network.

## 3. Regulatory Mechanisms of the MGBA-Mediated Immune–Metabolic–Endocrine Network in ASD

In recent years, the core regulatory mechanisms of the MGBA in the pathological progression of ASD have been gradually elucidated, covering multiple dimensions including dysregulated microbe–host co-metabolism, aberrant immune responses, HPA axis dysfunction, and impaired neural pathway conduction [39]. These pathways do not function independently; instead, they form an immune–metabolic–endocrine interactive regulatory network that collectively mediates the bidirectional crosstalk between the intestinal microecology and the central nervous system. The core mechanistic actions can be summarized as follows: ① Gut microbiota metabolites act as critical signaling molecules to regulate neurodevelopment and behavior. ② Disruption of intestinal barrier integrity triggers microbial antigen translocation and initiates systemic immune-inflammatory responses. ③ HPA axis dysfunction exacerbates neuroendocrine imbalance and abnormal behavioral phenotypes. ④ The vagus nerve and enteric nervous system form the anatomical basis for the transmission of intestinal signals to the central nervous system.

### 3.1. Gut Microbiota Dysbiosis Triggers Abnormal Metabolic Profiles

**Figure 3:**
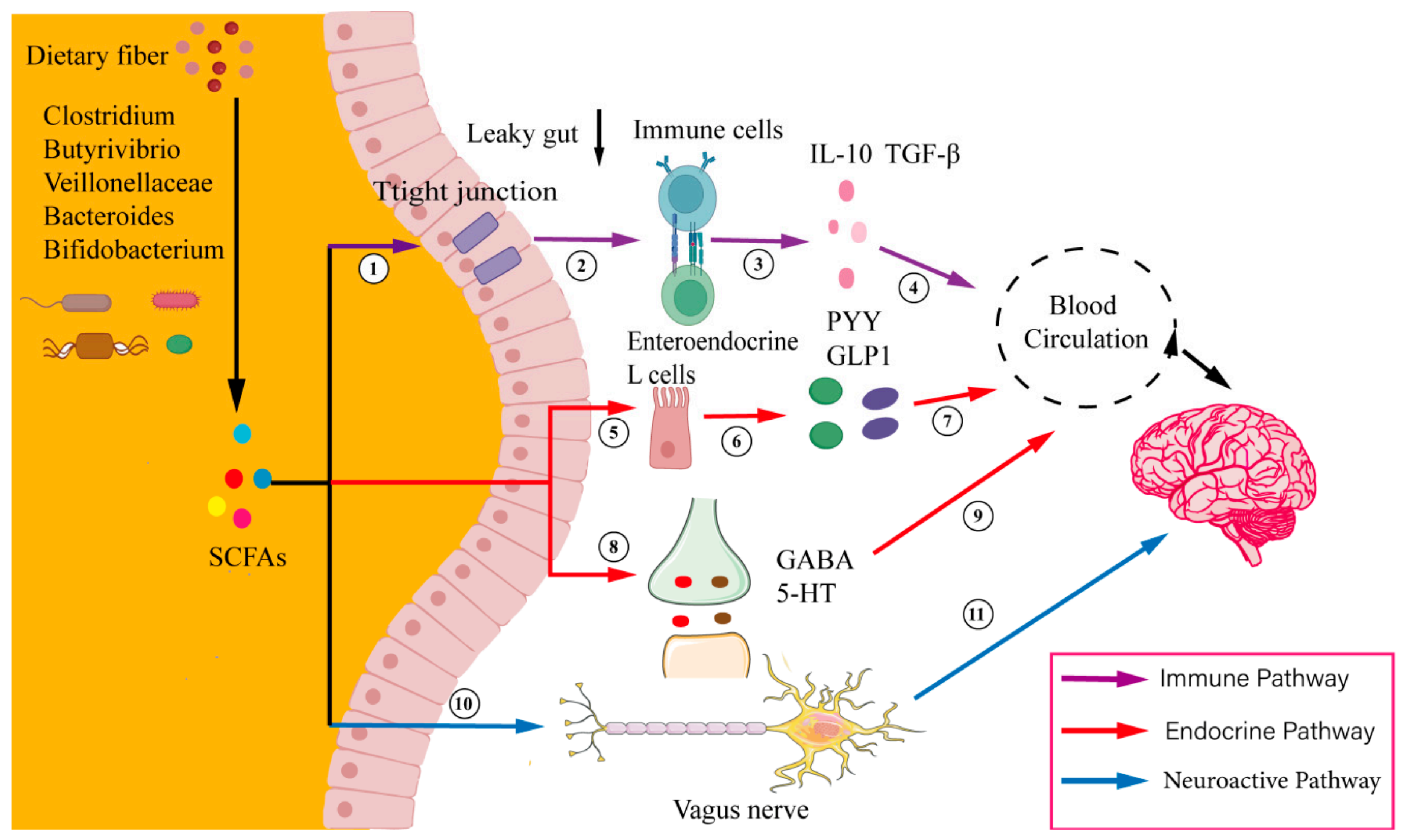
Mechanisms of SCFA-mediated GBA signaling. **Note:** (1) Immune Pathway (→): ① SCFAs regulate tight junction proteins; ② SCFAs cross intestinal epithelium; ③ immune cells release anti-inflammatory factors (e.g., IL-10); ④ anti-inflammatory factors enter circulation. (2) Endocrine Pathway (→): ⑤ SCFAs stimulate enteroendocrine L-cells; ⑥ L-cells secrete gut hormones (e.g., PYY); ⑦ gut hormones enter circulation; ⑧ SCFAs promote neurotransmitter release (e.g., GABA); ⑨ neurotransmitters enter circulation. (3) Vagal Pathway (→): ⑩ SCFAs directly activate vagus nerve; ⑪ neural signals transmitted to brain.

Structural disruption of the gut microbiota can directly alter the host’s co-metabolic profile, and the associated metabolites serve as the core chemical mediators for bidirectional signal transduction in the MGBA. Short-chain fatty acids (SCFAs) are the key metabolites produced by the fermentation of dietary fiber by gut microbiota, mainly including three monomers: acetic acid, propionic acid, and butyric acid. They participate in multiple processes such as intestinal barrier maintenance, immune regulation, endocrine signal transmission, and neurodevelopmental modulation. SCFAs bind to free fatty acid receptors on the surface of intestinal epithelial cells, induce enteroendocrine L cells to secrete glucagon-like peptide-1 (GLP-1) and peptide YY (PYY)—both of which are involved in the regulation of feeding behavior, cognitive function, and stress homeostasis [40]—and maintain intestinal barrier integrity by modulating tight junction protein expression (Figure 3).

Existing studies have demonstrated significant compositional imbalance of SCFAs in patients with ASD, and disrupted microbial metabolic profiles represent an important potential mechanism underlying ASD neuropathology [41]. The specific physiological functions of SCFAs are presented in Table 2 [42,43,44,45,46,47,48,49]. Notably, the pooled results from meta-analyses for individual short-chain fatty acid monomers indicate that there is no consistent global increase or decrease in SCFAs between children with ASD and neurotypically developed children. As a synthetic study, meta-analysis yields higher evidence strength than individual small-sample original research. SCFA measurements are highly dependent on sample sources, suggesting that ASD is not characterized by a simple overall reduction in SCFA levels, but by specific remodeling of the short-chain fatty acid profile [50]. Some studies have indicated a declining trend in total SCFA levels in patients with neuropsychiatric disorders, suggesting that insufficient protective metabolites may weaken the neuroprotective effects of SCFAs [51]. However, other studies have detected abnormally elevated overall SCFA levels in the serum and feces of patients with ASD, with the increase in propionic acid (PPA) being the most prominent. This alteration is closely correlated with the enrichment of PPA-producing bacteria such as *Clostridium* and *Bacteroides*, implying that PPA-producing taxa may serve as potential microbial biomarkers for ASD [52]. Mechanistic investigations by Frye et al. [53] have revealed that PPA participates in ASD pathogenesis by inducing mitochondrial dysfunction. Abdelli et al. [54] examined behavioral phenotypes in ASD mouse models and found that PPA exposure triggers excessive glial activation, which in turn causes neurological impairment. Genistein (GNT) can activate the neuronal cAMP/CREB/PKA pathway, reverse impaired mitochondrial function, suppress neuroinflammation, regulate inflammatory cytokine levels and the expression of autism-associated proteins, alleviate PPA-induced neurotoxicity, and improve behavioral phenotypes in ASD model mice [55]. These findings collectively suggest that PPA may act as a critical metabolic mediator linking gut microbiota dysbiosis to neuronal damage. Overall, the SCFA profile imbalance driven by gut microbiota dysbiosis is characterized by reduced levels of the protective monomers acetic acid (AA) and butyric acid (BTA), accompanied by excessive accumulation of the toxic monomer PPA. Together, these alterations constitute metabolic risk factors for the onset and progression of ASD.

In addition to SCFAs, multiple gut microbiota-derived small-molecule metabolites have been confirmed to participate in the pathological progression of ASD. For instance, in vitro cellular experiments have shown that p-cresol impairs dendritic development, synaptogenesis, and synaptic function in hippocampal neurons, suggesting that it may contribute to neurodevelopmental abnormalities by disrupting the assembly of neural circuits [56]. Furthermore, Needham et al. [57] detected significantly elevated 4EPS levels in the blood of mice colonized with gut microbiota from patients with ASD.

Lipoteichoic acid (LTA) is a major cell-wall component of Gram-positive bacteria and a natural ligand for TLR2. In vitro experiments have demonstrated that stimulation of primary monocytes from children with ASD by LTA elicits aberrantly elevated production of pro-inflammatory cytokines IL-1β, IL-6, and TNF-α [58]. At the genetic level, LTA activation induces the expression of immune-related genes, some of which are differentially regulated between ASD and typically developing control samples [59], suggesting that LTA may represent another important pathway linking gut dysbiosis to neuroinflammation in ASD.

Trimethylamine N-oxide (TMAO) is a small-molecule metabolite generated by gut bacterial metabolism of dietary choline. A small-sample anti-inflammatory dietary intervention study in children with ASD revealed that decreased plasma TMAO levels were strongly associated with cognitive improvements in verbal ability, attention and executive function. Network-based modeling indicated that ΔTMAO could serve as a candidate responsive biomarker for nutritional intervention in ASD [60]. Preclinical evidence from reviews in the field of mild cognitive impairment has shown that TMAO can trigger neuroinflammation via activation of the NLRP3 inflammasome and NF-κB signaling cascade [61]. Nevertheless, direct evidence is still lacking regarding whether this signaling axis also operates in ASD patients.

In addition, qPCR assays have detected significantly increased abundance of D-lactate dehydrogenase genes from Bacteroides fragilis and Alistipes finegoldii in the gut of children with ASD [62], implying enhanced potential for production of the neurotoxic metabolite D-lactate. Collectively, these findings indicate that multiple gut microbiota-derived bacterial products constitute a complex molecular network underlying MGBA-mediated pathological progression of ASD through multidimensional crosstalk among immune, metabolic and neural pathways. This metabolite, primarily produced by Firmicutes and Bacteroides ovatus, can enter the central nervous system via the systemic circulation, alter cerebral neural activity patterns, and induce anxiety-like and social deficit behaviors, further corroborating the core role of microbiota-derived metabolic dysregulation in ASD.

Collectively, the regulatory effects of gut microbiota metabolic disturbance on ASD do not depend on the independent action of a single molecule; instead, synergistic alterations of multiple classes of metabolites constitute a systematic metabolic risk network that drives neurodevelopmental abnormalities via mitochondrial damage, neuroinflammation, and intestinal barrier disruption. Notably, different metabolites can form complex regulatory networks through synergistic toxicity or compensatory antagonistic effects, which serves as an important metabolic basis for the high individual heterogeneity of ASD pathogenesis. Abnormal metabolic profiles further compromise intestinal barrier integrity, providing a pathological foundation for microbial antigen translocation and subsequent immune cascade activation.

### 3.2. Metabolic Dysregulation Synergistically Disrupts Intestinal Barrier Integrity

Structural dysbiosis of the gut microbiota and aberrant microbial metabolites directly impair the integrity of the intestinal epithelial barrier, leading to markedly increased intestinal permeability. This process represents a critical node for the systemic dissemination of intestinal signals. Only subsets of ASD patients exhibit elevated intestinal permeability, and detection rates vary substantially across different cohorts and detection methodologies. At present, direct functional-assay evidence regarding whether intestinal permeability is genuinely elevated in children with ASD remains controversial. Several studies employing the lactulose–mannitol ratio assay have reported no significant differences between children with ASD and control subjects [63]. Nevertheless, other investigations have documented abnormal intestinal permeability in pediatric ASD populations. Laura et al. [64]. assessed intestinal permeability using the lactulose–mannitol ratio and observed abnormal permeability in approximately one-third of children with ASD, whereas the proportion was substantially lower in control groups. Piras et al. [65] also indicated alterations in intestinal permeability among children with ASD based on urinary metabolomic analysis. In this context, Luo et al. [66] proposed that gut dysbiosis may trigger abnormal expression of intestinal mucosal structural proteins, impaired mucin synthesis and disruption of mucus-layer integrity, thereby compromising intestinal barrier function at the structural level and aggravating permeability injury.

At the molecular regulatory level, zonulin, a key endogenous protein governing intestinal tight junctions, increases intestinal permeability by inhibiting the assembly of the tight junction protein occludin, and is recognized as one of the core regulatory molecules underlying intestinal leakiness [67]. However, intestinal barrier disruption in ASD may occur via a zonulin-independent pathway. For example, clinical studies have detected no significant difference in serum zonulin levels between children with ASD and healthy controls, but serum occludin levels are significantly reduced, suggesting that tight junction disruption in ASD populations may involve a zonulin-independent molecular pathway [68]. Furthermore, upon increased intestinal permeability, microbial antigens such as lipopolysaccharide (LPS), a cell-wall component of intestinal Gram-negative bacteria, can translocate into the systemic circulation and trigger systemic immune-inflammatory responses. This induces the release of pro-inflammatory cytokines including IL-1β and IL-6, which further cross the blood–brain barrier to elicit central neuroinflammation, ultimately leading to ASD-related cognitive dysfunction [69]. Clinical cohort observations [70] have revealed that elevated plasma LPS levels are detectable in ASD subgroups with gastrointestinal comorbidities such as constipation, and this alteration can be reversed following microbial intervention. Notably, such phenomena are not observed in ASD individuals without gastrointestinal complications. Interventional studies have also confirmed that combined intervention with bee pollen and probiotics enhances intestinal barrier function by upregulating tight junction protein expression, ameliorates the intestinal leak phenotype, and improves behavioral abnormalities in ASD model mice [71]. These correlational observations support a hypothetical model in which intestinal barrier injury may initiate downstream immune-inflammatory cascades in subsets of individuals with ASD. Discrepancies across studies may stem from sample heterogeneity, detection methodologies, and variable severity of gastrointestinal symptoms among participants.

### 3.3. Intestinal Barrier Injury-Mediated Immune Activation and Neuroinflammation

Gut-associated lymphoid tissue (GALT) plays a pivotal role in bidirectional regulation via the MGBA. Microbial antigen translocation induced by elevated intestinal permeability further activates the mucosal immune system, stimulates intestinal plasma cells to secrete immunoglobulin A (IgA), and consequently triggers systemic inflammatory status [72]. Clinical observations have detected intestinal mucosal immune perturbations in ASD patients with gastrointestinal comorbidities, manifested as markedly elevated IgA levels [63], and significantly upregulated pro-inflammatory cytokines IL-1β, IL-6, and IL-8 in peripheral blood [73,74]. Epigenetic research further revealed hypomethylation of the promoter regions of IL-1β and IL-6 genes in peripheral blood from ASD patients, which serves as a critical molecular mechanism sustaining persistent high expression of pro-inflammatory factors and chronic inflammatory activation [75]. Notably, inflammation is not confined to peripheral tissues. Animal experiments have uncovered extensive immune dysfunction in the cerebellum, peripheral blood, bone marrow, spleen and other immune organs of ASD model mice, further supporting the tight correlation between aberrant immune inflammation and ASD behavioral phenotypes [76]. Tan et al. [77] detected elevated levels of inflammatory mediators including TNF-α, IL-4, IL-21 and B cell-activating factor (BAFF) in the cerebrospinal fluid (CSF) of ASD patients, directly confirming the existence of central neuroinflammation. Notably, fecal calprotectin serves as a standard biomarker for neutrophil-mediated acute intestinal inflammation. Nevertheless, Mathew et al. [78] demonstrated that elevated calprotectin is not a universal feature among children with ASD and is only observed in subgroups complicated by gastrointestinal comorbidities. This indicates that prominent intestinal inflammation dominated by neutrophil infiltration cannot be regarded as a common pathological feature of ASD. Even with normal calprotectin levels, some ASD patients still exhibit low-grade subclinical mucosal immune perturbations, and such abnormalities are highly restricted to subgroups with gastrointestinal symptoms. Based on these subgroup-specific intestinal barrier injuries and low-grade mucosal immune disturbances, gut-derived aberrant signals can further trigger peripheral immune activation and ultimately mediate imbalanced central neuroinflammation.

At the central nervous system level, excessive microglial activation represents the hallmark neuro-inflammatory feature of ASD. Studies have demonstrated that intestinal barrier damage allows microbial components and peripheral pro-inflammatory factors to enter the circulation and cross the blood–brain barrier, triggering imbalanced microglial polarization characterized by an overactivated pro-inflammatory M1 phenotype and impaired anti-inflammatory M2 function. This imbalance leads to aberrant synaptic pruning, neuronal damage and defective neural circuit development [77]. Consistent animal studies have reported region-specific increases in microglial density accompanied by prominent pro-inflammatory signatures in the brains of ASD model mice [79]. Furthermore, Tetreault et al. [80] observed not only increased microglial quantity but also typical activated morphological traits including enlarged cell bodies and shortened, thickened processes in post-mortem brain tissues from ASD patients. Intervention experiments targeting microglial activation showed that the ErbB4 inhibitor AG1478 suppresses excessive microglial activation by modulating the neuregulin 1 signaling pathway and alleviates behavioral deficits in ASD model mice, providing experimental evidence for neuroinflammation-targeted therapies [81]. Brain tissue studies have indicated that microglial activation and polarization imbalance may originate from critical windows of early neurodevelopment. Specifically, perinatal immune stress can induce persistent phenotypic reprogramming of microglia [82]. In addition, further brain tissue analyses have confirmed a significant increase in primed-state microglia in the temporal cortex of ASD patients, and this alteration is positively correlated with donor age, suggesting that abnormal microglial activation can persist into adulthood and even worsen with aging [83]. Nevertheless, due to the limitations of sample size and individual heterogeneity, the exact proportion and causal role of microglial polarization imbalance across different ASD subgroups remain difficult to clarify.

In addition, multiple studies utilizing the maternal immune activation (MIA) model have further validated that the MGBA participates in ASD pathogenesis through immune pathways. For example, Salia et al. [84] found that fecal microbiota transplantation from MIA mice elevates serum IL-4 and IL-7 levels and induces ASD-like behaviors in recipient animals, which offers solid evidence linking microbiota transfer to microglial activation. Zhang et al. [85] further demonstrated robust microglial hyperactivation in the prefrontal cortex of MIA offspring, which correlates with overexpression of the Na^+^-K^+^-Cl^−^ cotransporter (NKCC1); the NKCC1 inhibitor bumetanide (BTN) can rescue ASD-associated behavioral abnormalities.

**Figure 4:**
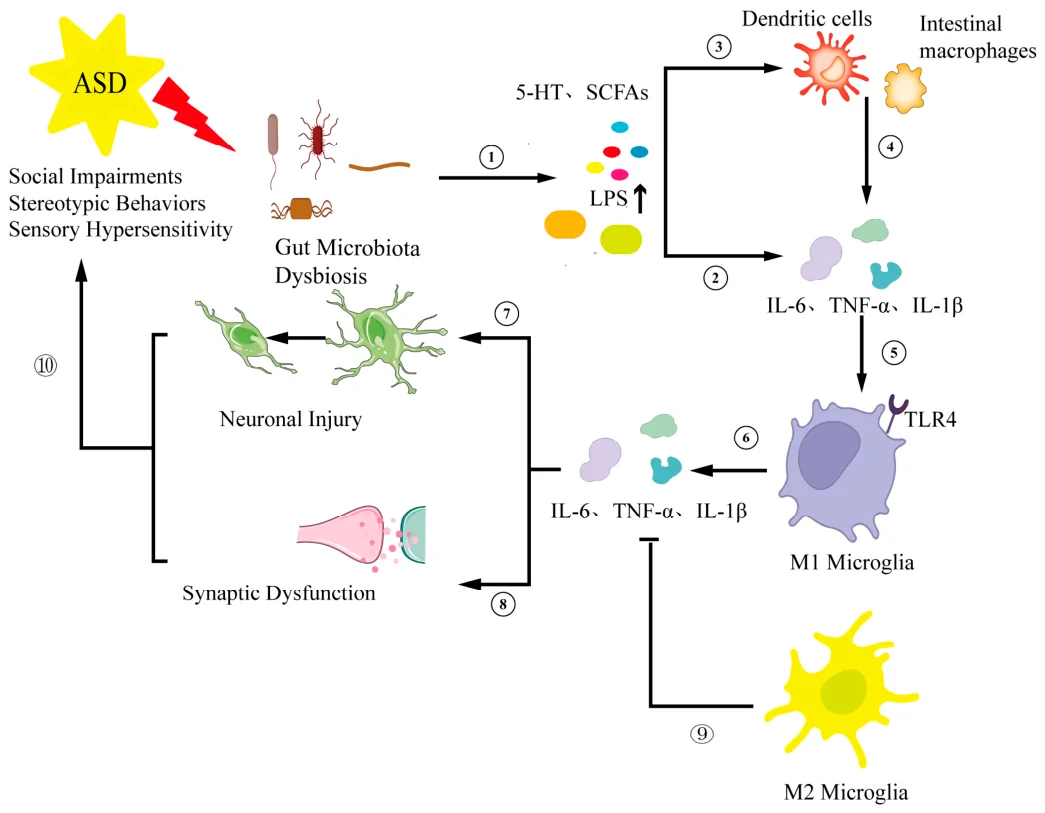
The mechanisms of microglial polarization imbalance and neuroinflammation in ASD. **Note:** ① Gut microbiota dysbiosis in ASD disturbs the production of microbial-derived metabolites and elevates LPS levels. ② Barrier-dependent route: Microbial products such as LPS translocate across the impaired intestinal barrier into systemic circulation to trigger peripheral inflammatory response. ③ Barrier-independent route: Microbial metabolites directly activate intestinal innate immune cells, without LPS translocation through damaged gut barrier. ④ Activated intestinal immune cells secrete pro-inflammatory cytokines. ⑤ These pro-inflammatory cytokines act on TLR4 and induce pro-inflammatory M1 microglial activation. ⑥ Activated M1 microglia further release abundant pro-inflammatory cytokines. ⑦ Excessive pro-inflammatory mediators induce neuronal injury. ⑧ Pro-inflammatory signals also lead to synaptic dysfunction. ⑨ M2-type microglia exhibit impaired immunoregulatory capacity and fail to exert sufficient anti-inflammatory effects. ⑩ Collectively, neuronal injury and synaptic dysfunction contribute to the core behavioral phenotypes of ASD. The above mechanistic conclusions are derived from animal model studies.

These findings suggest that the immune-inflammatory pathway serves as a core link connecting gut microbiota dysbiosis and central neurobehavioral abnormalities. Gut dysbiosis can disrupt intestinal barrier integrity, enabling microbes and their products to enter the systemic circulation, sequentially activating the peripheral immune system and central microglia, and triggering cascading amplified neuro-inflammatory responses that ultimately contribute to the onset and progression of ASD. Notably, intestinal barrier impairment is not a universal pathological feature of ASD. It has only been verified in a subgroup of children with ASD complicated by gastrointestinal symptoms [86], and consistent validation across the entire ASD population remains absent. Gut microbiota dysbiosis disrupts intestinal barrier integrity, facilitating the translocation of microbes and their metabolites into systemic circulation, which sequentially activates peripheral immunity and central microglia to initiate amplified neuro-inflammatory cascades that drive the onset and progression of ASD. Activated microglia exhibit severe M1/M2 polarization imbalance: overabundant M1 microglia secrete large quantities of pro-inflammatory cytokines (IL-1β, TNF-α) and reactive oxygen species to exert neurotoxicity, while M2 microglia lose sufficient anti-inflammatory and neuroreparative capacity. Dysfunctional microglia also disrupt physiological synaptic pruning, impairing the development and plasticity of neural circuits. Such structural and neuronal abnormalities ultimately manifest as the core clinical symptoms of ASD [87] (Figure 4). Sustained peripheral and central inflammatory signals further hyperactivate the HPA axis, which retrogradely remodels the intestinal microenvironment via neuroendocrine pathways and amplifies this vicious pathological cycle. Nevertheless, it is worth noting that intestinal barrier injury acts as an important upstream trigger for immune-neuro-inflammatory cascades in certain ASD subgroups; however, it is not an essential prerequisite.

### 3.4. Immune Signal Amplification and Endocrine Feedback Mediated by the HPA Axis

The hypothalamic–pituitary–adrenal (HPA) axis serves as the core regulatory axis of the neuroendocrine system, exerting a vital function in linking the intestinal microecology, systemic immunity and central neural circuits. Intestinal barrier injury induces chronic systemic inflammation, and such immune signals cross the blood–brain barrier to trigger excessive HPA axis activation. Persistently elevated cortisol conversely disrupts intestinal homeostasis and exacerbates neuroinflammation, forming a vicious pathological loop of the microbiota–intestine–immunity–HPA axis.

HPA axis dysfunction represents a prominent pathological hallmark of ASD, which forms an intimate interactive network with gut microbiota dysbiosis and aberrant immune inflammation. On one hand, gut microbiota and their metabolites such as SCFAs act as key peripheral regulators of HPA axis homeostasis. Animal experiments have validated that gut microbial community dysbiosis impairs the negative feedback loop of the HPA axis, resulting in neuroendocrine disorders and anxiety-like behavioral phenotypes [88]. Clinical investigations have also revealed widespread disruptions of cortisol secretion rhythm and abnormal stress responses in ASD patients, which are tightly correlated with alterations in gut microbiota composition. In a triple-blind randomized controlled trial of healthy volunteers, Boushra et al. [89] demonstrated that colon-targeted supplementation with SCFAs blunts cortisol responses to acute psychological stress. This indicates that microbial metabolites modulate HPA axis activity to maintain neuroendocrine homeostasis, providing indirect evidence for the involvement of microbiota–HPA axis crosstalk in ASD pathogenesis. At the molecular level, sodium butyrate, a histone deacetylase inhibitor, can restore HPA axis homeostasis and alleviate anxiety-like behaviors in ASD animal models by epigenetic regulation of stress-related genes. On the other hand, the HPA axis forms a closed-loop regulatory network with the immune system and gut microbiota [90]. On the other hand, the HPA axis forms a closed-loop regulatory network with the immune system and gut microbiota. HPA axis dysfunction disturbs the rhythmic secretion of cortisol, which mediates aberrant immune cell activation via glucocorticoid receptors and facilitates peripheral and central neuroinflammation. Neuroinflammation further activates microglia to release pro-inflammatory cytokines including IL-1β and IL-6, aggravating neuronal damage and synaptic dysfunction. Meanwhile, gut microbiota dysbiosis modulates HPA axis activity through metabolites such as SCFAs. Excess cortisol in turn impairs the integrity of intestinal tight junctions, alters intestinal secretion and peristalsis, remodels the compositional and metabolic profile of gut microbiota, and further aggravates microecological imbalance. This ultimately generates a vicious cycle of “microbiota dysbiosis-HPA axis activation-barrier damage-worsened microbiota dysbiosis” that collectively drives the pathological progression of ASD [88].

In conclusion, dysregulated HPA axis function participates in the biological onset and progression of ASD-associated manifestations including anxiety, irritability and impulsivity through multiple pathways such as neuroendocrine modulation, the amplification of immune-inflammatory responses, and the remodeling of gut microecology. As a critical hub connecting central neural function and the peripheral intestinal microenvironment, the HPA axis also provides important neuroendocrine targets for targeted ASD interventions.

### 3.5. Neural Regulation of the MGBA Mediated by the Vagus Nerve and Enteric Nerves

The neural conduction pathways of the MGBA are anchored by the vagus nerve and enteric nervous system (ENS), serving as a direct route for rapid bidirectional signal exchange between the gut and brain, and functionally complement immune and endocrine pathways. As the primary neural trunk for gut–brain communication, the vagus nerve undertakes both afferent and efferent signal transmission. The ENS, a local neural network embedded within the intestinal wall, can independently regulate physiological processes including intestinal motility, secretion, and local immune responses.

Current evidence indicates that structural and functional abnormalities of the ENS constitute the core pathological basis for the high prevalence of gastrointestinal comorbidities in ASD, and act as a critical local node linking gut microbiota dysbiosis to central neurodevelopmental defects, with bidirectional regulatory interactions between the two. On one hand, gut microbiota dysbiosis directly triggers structural remodeling of the ENS. For instance, Jacques et al. [91] transplanted fecal microbiota from ASD patients into wild-type mice, and observed prominent structural reconstruction of the intestinal ENS in recipient animals, accompanied by downregulated neural adhesion molecule expression and disrupted intestinal motility and secretion. This demonstrates that microbiota dysbiosis mediates gastrointestinal symptoms and aberrant gut–brain signaling via ENS impairment. On the other hand, ASD-associated genetic mutations can primarily induce ENS dysfunction. Hosie et al. [29] found that synaptic impairment of the ENS emerges prior to gut microbiota dysbiosis in mutant mice carrying autism-related genes, alongside gastrointestinal dysmotility. This suggests that congenital neurodevelopmental defects in ASD alter intestinal peristalsis and mucus secretion patterns, reshape the luminal microenvironment, and selectively enrich pathogenic bacterial taxa. Collectively, ENS damage and microbiota dysbiosis form a vicious local cycle: genetically driven primary ENS defects induce dysbiosis, which in turn exacerbates structural and functional deterioration of the ENS.

Furthermore, the vagus nerve represents the most direct conduit for transmitting gut microbial signals to the central nervous system, and plays an irreplaceable role in microbiota-mediated modulation of social and emotional behaviors. Studies in ASD models have shown that supplementation with *Lactobacillus* reuteri markedly rescues social deficits in mice, whereas this therapeutic effect is completely abolished following subdiaphragmatic vagotomy, verifying that the behavioral regulatory effects of probiotics rely on intact vagal signaling [92]. Consistent with these findings, Bravo et al. [93] reported that *Lactobacillus rhamnosus* ameliorates emotional behavioral phenotypes by upregulating vagal γ-aminobutyric acid (GABA) receptor expression, an effect also dependent on vagal integrity. Notably, vagal regulation is bidirectional: efferent fibers originating from the brain modulate intestinal smooth muscle contraction, mucus secretion and epithelial barrier function to remodel the gut microenvironment, thereby retroactively shaping the colonization and composition of gut microbiota. This efferent regulatory mechanism largely accounts for the phenomenon whereby stress and emotional disturbances aggravate microbial dysbiosis.

In summary, neural pathways centered on the vagus nerve and ENS are indispensable components of the MGBA regulatory network, jointly mediating bidirectional crosstalk between the intestinal microecology and central nervous system. Nevertheless, the molecular targets and signaling cascades through which gut microbiota modulate enteric and vagal nerves remain incompletely characterized, and the synergistic regulatory effects across multiple pathways require systematic elucidation in future research.

## 4. Neurotransmitter Dysregulation Mediated by the MGBA and Its Pathological Roles in ASD

Neurotransmitters serve as the core chemical messengers in the bidirectional communication network of the MGBA, directly governing neural circuit development and ASD behavioral phenotypes. Gut microbiota disrupt peripheral and central neurotransmitter homeostasis via multiple approaches including metabolic modulation, modification of enzyme expression, and precursor supply [94]. The key signaling molecules fall into two major categories: monoamines (5-hydroxytryptamine (5-HT), dopamine (DA), histamine) and inhibitory amino acids (γ-aminobutyric acid (GABA)) [24,95]. Focusing on these four critical neurotransmitters, this section reveals the immune–metabolic–endocrine interactive regulatory mechanisms from a neurochemical perspective and explores microecology-targeted intervention candidates for ASD.

### 4.1. Gut Microbiota-Regulated 5-HT Dysbiosis Induces Neurodevelopmental Abnormalities

5-HT participates in central nervous system maturation, formation of social behaviors, and emotional homeostasis. Relying on metabolites such as SCFAs, gut microbiota modulates the activity of tryptophan hydroxylase 1 (TPH1) expressed in host enterochromaffin cells, which determines the synthesis efficiency of peripheral 5-HT. Multiple studies have validated that gut microbiota dysbiosis associated with ASD triggers 5-HT system dysfunction. For example, Tian et al. [96] observed markedly downregulated TPH1 expression and reduced peripheral 5-HT concentrations in colonic tissues of germ-free mice; colonization with specific microbiota or SCFA supplementation restored the 5-HT synthetic capacity of enterochromaffin cells. Animal experiments demonstrated that elevated peripheral 5-HT induces ASD-like social deficits in mice, while knockout of the 5-HT transporter to strengthen 5-HT reuptake substantially alleviates social impairment in model animals, indicating that aberrant 5-HT transport and reuptake represent a vital molecular mechanism underlying ASD [97]. Furthermore, Yang et al. [98] verified that disrupted 5-HT signaling not only directly disturbs neurogenesis and synaptic plasticity but also indirectly modulates ASD behavioral phenotypes through regulating immune-inflammatory responses. Nevertheless, the precise mechanisms by which the 5-HT pathway alters central neural function remain incompletely elucidated, requiring integrated multi-omics research to refine theories for targeted interventions.

### 4.2. Region-Specific Dysregulation of the GABAergic System Mediates Imbalanced Neuronal Excitation/Inhibition

As the predominant inhibitory neurotransmitter in the central nervous system, GABA sustains the excitation/inhibition (E/I) balance of neural circuits. Accumulating evidence has revealed prominent regional, hemispheric, and gender heterogeneity in GABA levels among patients with ASD, and aberrant GABAergic signaling is widely recognized as one core pathophysiological mechanism of ASD. For instance, Umesawa et al. [99] measured regional GABA concentrations in ASD patients via magnetic resonance spectroscopy, and identified significant GABA reduction solely in the supplementary motor area (SMA), which positively correlated with sensory hypersensitivity. A study on gender heterogeneity conducted by Fung et al. [100] demonstrated sex-specific correlations between GABA concentrations in the thalamus and prefrontal cortex and autism spectrum quotient (AQ): negative correlation was observed in males, whereas positive correlation existed in females. Saleh et al. [101] further investigated hemispheric GABA asymmetry in children with ASD and reported elevated GABA levels in the right temporal lobe with no obvious alterations in the left hemisphere, highlighting the complexity and spatial specificity of GABAergic pathology in ASD. Beyond altered neurotransmitter concentrations, abnormal GABA receptor expression also attenuates inhibitory neural transmission. Tiffany et al. [102] detected downregulated expression of the α2 subunit of GABAA receptors at the axon initial segment of pyramidal neurons in the prefrontal cortex of ASD patients, which directly impairs inhibitory signaling and disrupts neural circuit E/I balance. In addition, Tabouy et al. [103] found that supplementation with Lactobacillus reuteri upregulates central GABA receptor expression and rescues ASD-like behaviors in mouse models. This finding further confirms that gut microbiota modulate central GABA homeostasis through multiple pathways including the vagus nerve, microbial metabolites, and immune inflammation, offering novel strategies for neural circuit-targeted interventions. Preclinical evidence from non-ASD experimental models supports associations between gut microbiota, dopamine reward circuit and addictive-like behaviors. Nevertheless, this review focuses primarily on social reward deficits and stereotyped behaviors relevant to ASD. Direct translational evidence linking this signaling pathway to addictive phenotypes in human ASD populations remains absent, which represents a promising direction for future research.

### 4.3. Gut Microbiota-Derived Histamine Dysregulation Synergistically Induces Immune Activation and Neural Injury

Histamine functions as both an immune mediator and neurotransmitter, and gut microbiota-derived histamine is recognized as one of the critical chemical messengers mediating bidirectional MGBA communication. Existing evidence indicates that microbiota-originated histamine participates in ASD pathogenesis, and disrupted histamine metabolism acts as a distinctive biochemical biomarker of ASD. First, Sònia et al. [104] found that multiple intestinal commensal genera such as *Staphylococcus* and *Proteus* synthesize histamine through histidine decarboxylase catalysis, suggesting that microbiota-derived histamine may drive ASD progression via the MGBA. On this basis, Giada et al. [105] further validated that excess microbiota-produced histamine triggers neurological dysfunction through receptor-dependent pathways. In addition, clinical research by Rashaid et al. [106] revealed that plasma histamine concentrations in children with ASD are up to 5.3-fold higher than those in healthy controls, demonstrating excessive activation of the histaminergic system as a core biochemical feature of ASD. This conclusion is further supported by studies using ASD animal models and brain transcriptome analysis. For instance, Fu et al. [107] confirmed that abnormal abundance of histamine-producing gut bacteria disrupts central neurotransmitter networks in ASD model mice. Transcriptomic profiling of brain tissues also detected dysregulated expression of histamine-related gene sets in ASD patients, which amplifies pathological damage by stimulating neuroinflammation [108].

Histamine elicits diverse biological responses by binding to distinct histamine receptor subtypes. Karthikkumar et al. [109] reported that the histamine H3 receptor (H3R) mediates negative feedback inhibition of histamine release, and the H3R antagonist ST-713 effectively rescues ASD-like behavioral deficits in model mice, indicating H3R as a potential therapeutic target for ASD. Notably, the histaminergic system undergoes extensive crosstalk with other neurotransmitter systems including 5-HT, GABA and dopamine, jointly forming an intricate neurochemical disorder network underlying ASD [110].

### 4.4. Intestinal Microbiota-Regulated Dopaminergic Abnormalities Mediate Reward and Social Behavioral Disorders

As a key monoamine neurotransmitter, dopamine (DA) constitutes the mesocorticolimbic dopaminergic system and participates extensively in the regulation of cognitive function, social behaviors, and reward circuits. In the pathological context of ASD, gut microbiota dysbiosis leads to functional imbalance of the dopaminergic system and disrupts the normal regulation of reward and social behavioral circuits. Current studies have demonstrated that gut microbiota-derived fatty acid amides (FAAs) indirectly modulate central dopaminergic function through the MGBA. For instance, Dohnalová et al. [111] found that FAAs produced by intestinal commensal bacteria including *Eubacterium rectale* and *Coprococcus comes* activate the enteric nervous system. The signals are transmitted via the vagus nerve and enhance DA release in the ventral striatum, confirming that gut microbiota remotely modulates central dopaminergic circuits.

Dopaminergic abnormalities in ASD exhibit substantial heterogeneity across disease subtypes, age groups, and brain regions. Animal studies have shown that specific knockout of caspase-3 in dopaminergic neurons induces DA depletion and typical ASD-like behavioral phenotypes [112]. However, clinical evidence regarding the association between dopaminergic function and ASD remains inconsistent. Schalbroeck et al. [113] detected no significant differences in striatal dopamine synthesis capacity between ASD patients and healthy controls, further indicating the heterogeneous nature of dopaminergic dysfunction in ASD.

Despite such clinical heterogeneity, dopamine receptors are still considered potential therapeutic targets for ASD. Watson et al. [114] illustrated that D2-like receptors (including D2, D3, and D4 subtypes) maintain neuromodulatory network homeostasis by exerting negative feedback control over dopamine release and neuronal excitability. Pharmacological experiments by Takeuchi et al. [115] further demonstrated that D2/D3 receptor antagonists ameliorate social deficits in ASD model mice, providing experimental evidence for dopaminergic-targeted interventions. Accumulating mechanistic studies have clarified that peripheral DA cannot penetrate the blood–brain barrier; therefore, gut microbiota regulate central DA function only through indirect pathways involving the ENS, vagus nerve, and immune inflammation. The full molecular regulatory network governing dopamine and its receptors in ASD remains incompletely elucidated. Further characterization of subtype-specific dopaminergic alterations will help identify precise targets for ASD intervention.

## 5. MGBA-Based Intervention Strategies for ASD

**Figure 5:**
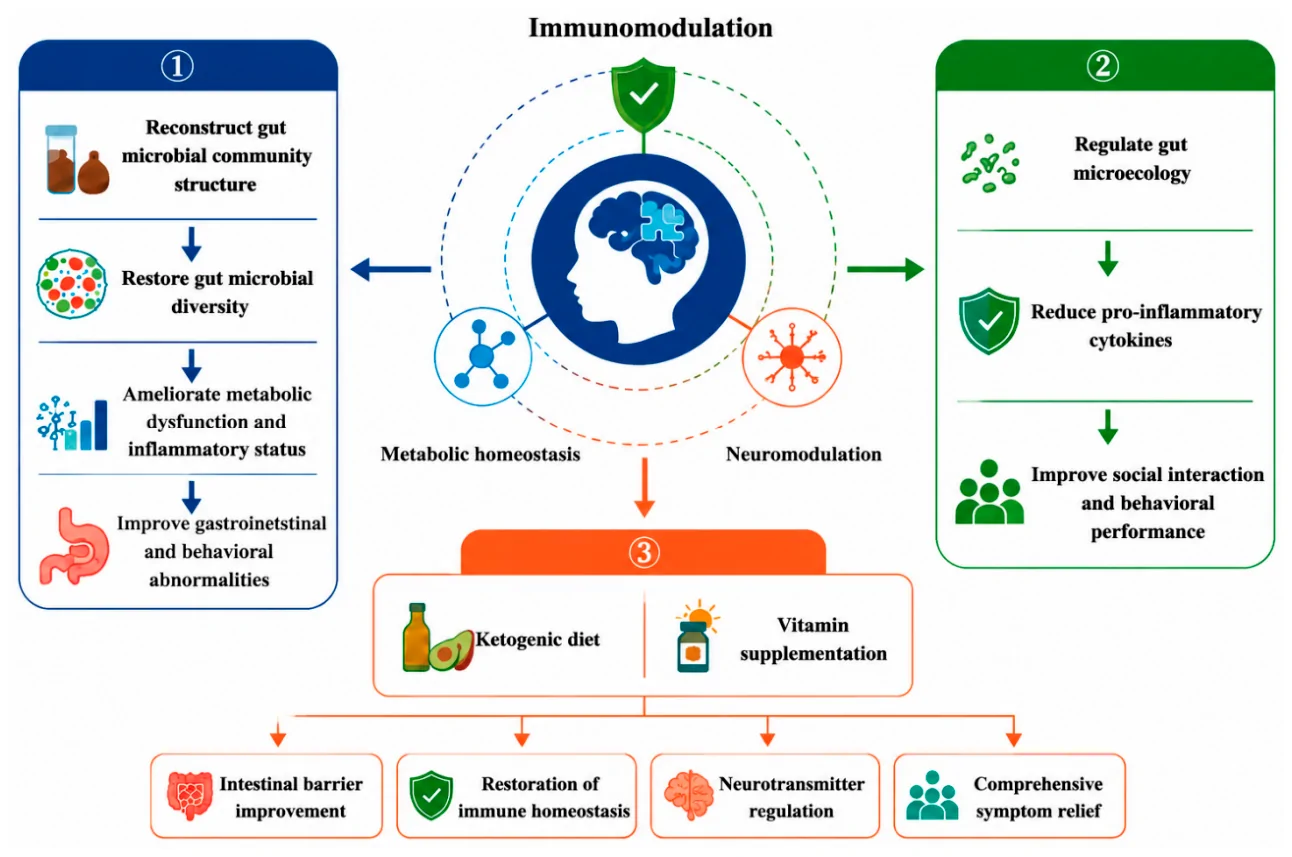
Intervention strategies for ASD based on the MGBA. **Notes:** ① Fecal microbiota transplantation (FMT): restructures gut microbial composition and restores microbial diversity to ameliorate systemic metabolic disorders, inflammatory responses, gastrointestinal symptoms and behavioral abnormalities. ② Probiotic intervention: modulates intestinal microecology, reduces pro-inflammatory cytokines, and improves social and behavioral performance in individuals with ASD. ③ Dietary intervention: consists of ketogenic diet and vitamin supplementation, which comprehensively relieve symptoms by regulating intestinal barrier integrity, immune homeostasis and neurotransmitter balance.

Traditional therapeutic interventions for ASD primarily target central neural pathways. In contrast, novel MGBA-targeted strategies focus on correcting upstream intestinal microecological dysbiosis. By blocking the pathological cascade spanning microbiota–metabolism–immunity–endocrine signaling, such interventions simultaneously alleviate gastrointestinal comorbidities and core neurobehavioral symptoms in children with ASD. Current mainstream MGBA-based approaches mainly include fecal microbiota transplantation (FMT), dietary modulation, and probiotic supplementation (Figure 5).

Currently, FMT remains in the clinical research stage. Several open-label clinical observations suggest that FMT can modulate the gut microecology of children with ASD, ameliorate metabolic and inflammatory phenotypes, and yield improvements in both gastrointestinal symptoms and core behavioral manifestations. Some follow-up observations further indicate long-term beneficial effects. For instance, Kang et al. [116] confirmed that FMT facilitates the colonization of beneficial bacteria such as Bifidobacterium and effectively relieves gastrointestinal symptoms. Further studies have shown that FMT remodels plasma and fecal metabolic profiles [117], modulates serum chemokines to alleviate systemic inflammation [118], and sustains behavioral improvements for at least two years of follow-up, indicating durable therapeutic effects [119]. At present, FMT remains at the experimental research stage and has not become a routine clinical therapy for ASD. One randomized, double-blind, placebo-controlled trial reported that FMT failed to significantly improve overall clinical symptoms of ASD and only showed limited positive effects in the social domain [120]. Most supportive clinical observations originate from open-label trials without placebo controls. Considering its invasive nature and complex multi-cycle procedural regimen, substantial placebo effects cannot be ruled out. Major translational bottlenecks include the absence of unified donor screening criteria, standardized operational protocols, and large-sample randomized controlled evidence. Notably, definite safety risks are associated with FMT. A meta-analysis reported that the overall incidence of FMT-related adverse events reaches 28.86%. The most frequent adverse events include gastrointestinal manifestations such as abdominal pain, diarrhea, bloating, vomiting and nausea [121]. A systematic review statement [122] indicated that routine clinical indications for FMT are restricted to moderate-to-severe or recurrent *Clostridioides* difficile infection. Accordingly, fecal microbiota transplantation, together with probiotic supplementation and ketogenic diet, should be categorized as exploratory microbiota-targeted intervention strategies, rather than a primary or preferred therapy for gut–brain axis disturbances in ASD.

Ketogenic diet (KD) represents a typical high-fat, low-carbohydrate dietary intervention. Current evidence from small-sample clinical trials and case observations indicates that KD ameliorates behavioral abnormalities in a subset of children with ASD, although it has not been established as a routine first-line clinical intervention. Christopher et al. [123] found that a high-fat and low-carbohydrate dietary pattern reverses the characteristic *Firmicutes*/*Bacteroidetes* ratio imbalance in ASD. Olson et al. [124] further demonstrated that KD enhances intestinal butyrate production, thereby exerting anti-inflammatory effects and protecting blood–brain barrier integrity. However, constipation is an extremely common side-effect of KD treatment, with an incidence rate ranging from 15% to 63%, especially among children [125]. It is mainly attributed to insufficient dietary fiber and fluid intake caused by the diet. Therefore, when implementing KD for children with ASD, close monitoring as well as active prevention and management of constipation are mandatory, such as ensuring adequate water intake and increasing vegetable consumption within dietary allowances. In addition, KD may also induce other adverse effects including vomiting, lethargy and hunger. Furthermore, its strict dietary regimen, poor patient compliance, lack of unified implementation criteria and long-term safety data limit its clinical application [126]. Supplemental vitamin A and vitamin D can serve as adjuvant strategies for KD. Recent studies have verified that vitamin A regulates neuronal differentiation and epigenetic modification [127], while vitamin D modulates 5-HT metabolism and suppresses chronic inflammation [128]. Both vitamins effectively rescue behavioral deficits and improve language performance in ASD models.

Probiotic intervention regulates intestinal homeostasis through the colonization of beneficial strains such as Bifidobacterium and Lactobacillus, featuring high safety, feasibility, and compliance. Multicenter randomized controlled trials have confirmed that supplementation with Lactobacillus and Bifidobacterium enhances social competence, reduces pro-inflammatory cytokine levels, and mitigates central neuroinflammation in children with ASD. However, inconsistent strain selection, undefined dosage and treatment courses, and substantial interindividual response variability remain major limitations [129,130]. In summary, the three categories of MGBA-targeted interventions block the multi-system pathological crosstalk from the peripheral microecological perspective, providing innovative non-central therapeutic strategies for ASD. Probiotic interventions can exert effects by altering microbiota-derived metabolites that influence brain function. For instance, in Shank3-knockout ASD mouse models, treatment with Lactobacillus reuteri significantly modulates gene expression and protein levels of GABA receptors in multiple brain regions, accompanied by ameliorated social behavioral deficits [103]. In addition, review-level evidence indicates that probiotics can affect biosynthetic pathways of neurotransmitters including 5-HT, GABA and dopamine via production of neuroactive metabolites such as SCFAs, thereby improving neurochemical homeostasis [131]. Furthermore, compared with emerging interventions such as the ketogenic diet and FMT, the evidence base for probiotics in ameliorating ASD is more well-established. Several meta-analyses focusing on probiotic therapy for children with ASD have been published, and pooled clinical trial data have demonstrated that probiotics can effectively alleviate partial gastrointestinal symptoms and behavioral abnormalities in ASD [132,133]. In contrast, most other intervention strategies rely on small-sample open-label trials, from which placebo effects cannot be readily excluded. Nevertheless, the absence of standardized clinical protocols and unclear mechanisms underlying individualized treatment responses remain critical obstacles limiting clinical translation [134].

## 6. Discussion

In recent years, the MGBA theory has broken through the limitations of single central nervous system perspectives in ASD research, and the association between gut microbiota dysbiosis and atypical ASD behaviors has been widely validated. However, most existing studies focus on unidirectional mechanistic interpretation, lacking a systematic summary of the bidirectional regulatory crosstalk among immune, metabolic, and endocrine networks. By integrating the latest research advances, this study constructs a bidirectional pathological regulatory model of MGBA-mediated ASD pathogenesis, compensating for the fragmented mechanistic descriptions in the current literature.

### 6.1. Key Research Gaps

Despite accumulating evidence supporting the pathological significance of immune, metabolic and endocrine interactive networks within the MGBA in ASD, several critical causal chains remain insufficiently validated and deserve explicit emphasis.

First, gut microbial alterations summarized in this review mainly focus on bacterial communities, while fungal components receive markedly limited attention. Several preliminary investigations have addressed this issue in recent years. A systematic review by Hu et al. [135] indicated that gut fungi exert non-negligible effects in neurodevelopmental disorders including ASD, and expansion of Candida constitutes a consistent feature of fungal dysbiosis. Zeng et al. [136] further highlighted that not only bacteria but also gut viromes and mycobiomes undergo substantial perturbations, suggesting that multi-kingdom microbial communities may act synergistically in ASD pathogenesis.

Second, the bidirectional vicious-cycle hypothesis proposed in this review has not yet undergone rigorous temporal validation in human longitudinal cohorts. Specifically, clear causal-temporal evidence is still lacking to determine whether gut dysbiosis emerges prior to intrauterine or early-postnatal neurodevelopmental abnormalities, or instead represents a secondary consequence of ASD-related behaviors and selective dietary patterns. Zhou et al. [137] performed bidirectional Mendelian randomization (MR) analyses to test causal associations in both directions: “microbiota-to-ASD” and “ASD-to-microbiota”. Their work provides genetic-epidemiological evidence supporting bidirectional causal links between gut dysbiosis and ASD. Nevertheless, the shortage of longitudinal cohort studies remains an important limitation within this field.

Third, the molecular mechanisms underlying intestinal barrier disruption remain incompletely characterized. Although zonulin serves as one of the key molecules regulating tight junctions, clinical studies on zonulin measurements in ASD have yielded considerably inconsistent findings. This indicates that zonulin-independent pathways may contribute to intestinal barrier injury. Eroğlu et al. [138] measured serum levels of five tight junction proteins among children with ASD and found no statistically significant differences for any of the five proteins between the ASD group and control group. This further suggests that intestinal barrier disruption in ASD may involve molecular pathways independent of zonulin, and that zonulin measurement alone cannot comprehensively evaluate the pathological status of intestinal permeability. The exact molecular cascade that drives elevated intestinal permeability in ASD remains to be elucidated.

Fourth, the causal status of imbalanced M1/M2 microglial polarization remains unclear. Although phenotypes featuring excessive M1-type activation and impaired M2-type function have been repeatedly observed in ASD animal models, longitudinal evidence is still lacking to determine whether such polarization imbalance acts as a driving factor for neuronal injury or merely represents a secondary concomitant phenomenon following neuropathological alterations. While pharmacological interventions targeting microglia have demonstrated certain therapeutic effects in animal models, substantial challenges persist for their clinical translation. Uncertainty regarding causal relationships constitutes one of the core obstacles.

Fifth, population heterogeneity and the shortage of validated biomarkers severely restrict clinical translation in this field. Inconsistent abundance results for microbial genera such as Lactobacillus across different cohorts adequately illustrate that factors including geographic distribution, dietary habits, age structure and genetic background exert profound influences on study outcomes. To date, no clinically validated combinatorial biomarkers consisting of gut microbes or metabolites are available for ASD subtyping or for predicting individual therapeutic responses. This represents a major bottleneck for MGBA-targeted interventions from the perspective of precision medicine.

Sixth, although microecological interventions represented by fecal microbiota transplantation show certain therapeutic potential, the vast majority of supporting evidence comes from small-sample open-label trials, preliminary pilot studies or animal models. Critical questions including donor screening criteria, strain specificity, optimal administration regimens, long-term safety, and effect durability over more than two years of follow-up remain unresolved. Ye et al. [139] performed fecal microbiota transplantation among 328 ASD patients with gastrointestinal symptoms and conducted follow-up for up to 60 months. Both behavioral symptoms and gastrointestinal function improved after intervention. Nevertheless, mild adverse events such as abdominal distension, vomiting, diarrhea and fever still occurred, indicating that further optimization of standardized protocols and accumulation of high-quality evidence-based medical evidence are still required in this field.

Taken together, these six inter-related and mutually restrictive research gaps constitute major barriers that hinder the transition from mechanistic interpretation to clinical translation within the MGBA-ASD research field. Future research requires coordinated advances in multiple directions, including the construction of standardized multicenter cohorts, multi-omics integrative analyses, and rigorously designed randomized controlled trials.

### 6.2. Integrated View of MGBA-Driven ASD Pathological Circuits

Gut microecological imbalance acts as a peripheral initiating factor for MGBA dysfunction and contributes to a cascading and self-amplifying pathological closed loop. Gut dysbiosis initially induces SCFA imbalance and accumulation of harmful metabolites, thereby disturbing neural energy metabolism and neurological function [140,141]. It further disrupts intestinal barrier integrity, triggers intestinal leakage, facilitates the entry of microbial antigens such as LPS into the bloodstream [142], activates peripheral inflammatory cascades, and ultimately induces excessive microglial activation and central neuroinflammation [143]. These peripheral signals can directly modulate central nervous system function via the vagus nerve and enteric nervous system, while persistent HPA axis activation leads to dysregulated cortisol secretion. Abnormally elevated cortisol reversely impairs intestinal homeostasis and reshapes microbiota composition, ultimately forming a self-amplifying pathological cycle characterized by dysbiosis, metabolic disturbance, immune activation, and endocrine imbalance.

**Figure 6:**
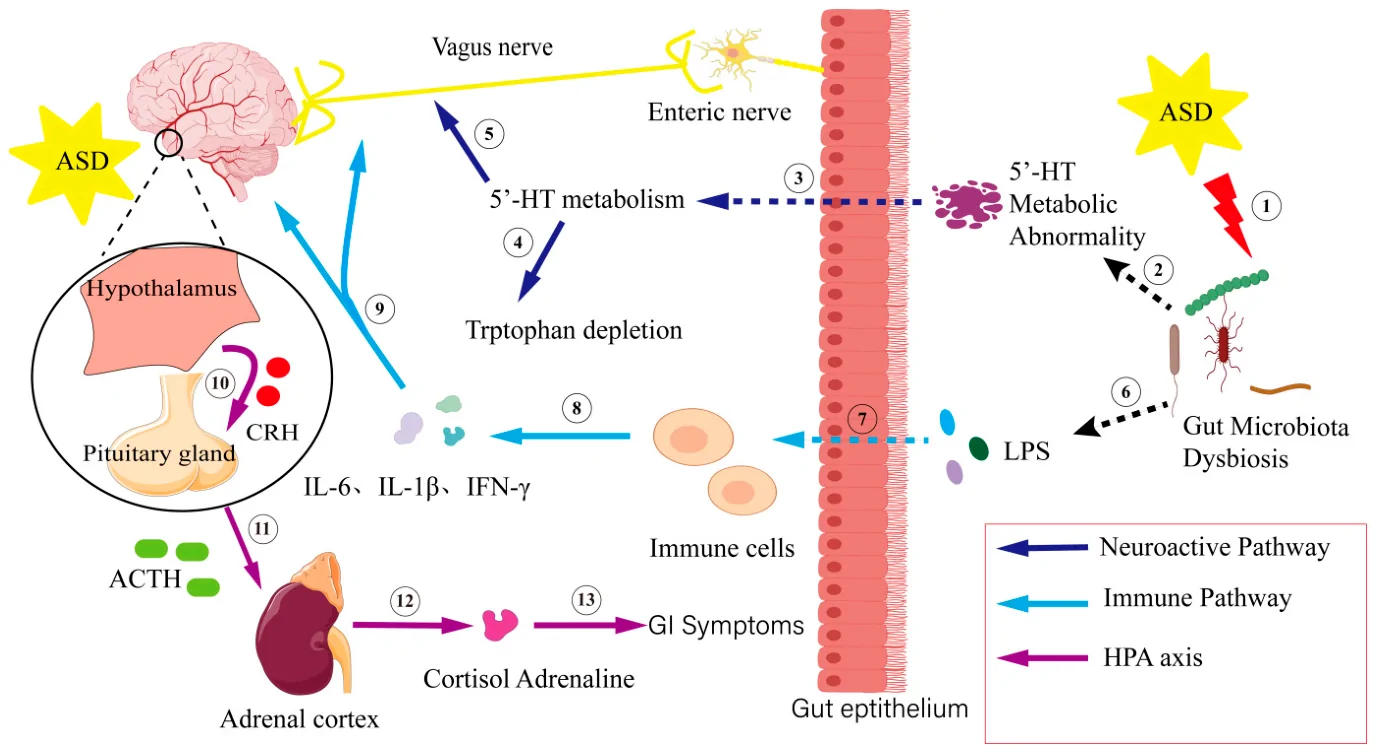
The core mechanistic roles of the microbiota–gut–brain axis in ASD. **Note:** (1) Neuro Pathway (←): ① ASD-related factors stimulate gut microbiota; ② gut microbiota metabolically produce 5-HT; ③ 5-HT crosses the intestinal mucosal epithelium; ④ 5-HT metabolism leads to tryptophan depletion; ⑤ signals are transmitted to the vagus nerve. (2) Immune Pathway (←): ⑥ Gut microbiota release LPS; ⑦ LPS activates immune cells; ⑧ immune cells release pro-inflammatory cytokines; ⑨ inducing neuroinflammation and altering brain functional states. (3) HPA Axis (←): ⑩ Hypothalamus secretes CRH, stimulating pituitary release of ACTH; ⑪ ACTH drives adrenal glands; ⑫ adrenal glands secrete cortisol; ⑬ stress hormones including cortisol feedback to exacerbate intestinal dysfunction.

Based on these pathological features, this review summarizes three core crosstalk pathways by which MGBA regulates ASD pathogenesis. The neural conduction pathway mediates bidirectional regulation between gut microbial metabolites and central neurotransmitters through the gut–brain neural circuit. The immune-inflammatory pathway realizes the cascade amplification and reverse feedback of gut-derived peripheral inflammation toward central neuroinflammation. As a core crosslinking node, the HPA axis endocrine pathway mediates bidirectional interactions between intestinal aberrant signals and systemic homeostasis disorders, collectively contributing to the pathological behavioral phenotypes of ASD (Figure 6).

## 7. Conclusions

In conclusion, this study systematically constructs a bidirectional interactive regulatory system of MGBA-mediated ASD pathogenesis and clarifies the synergistic mechanisms of the three core pathways, providing solid theoretical support for future ASD mechanistic research. Peripheral microecological interventions, represented by probiotics, FMT, and ketogenic diet, can correct metabolic disorders, inhibit neuroinflammation, and restore neurotransmitter homeostasis through multi-target regulation. Different from traditional central-targeted therapies, these strategies provide a novel perspective for the diagnosis and treatment of ASD. Future studies should unify detection standards and conduct large-scale stratified clinical trials to clarify the applicable population and safety of various microecological interventions. Multi-omics approaches and germ-free animal models are required to identify key crosslinking targets and elucidate the causal and temporal relationships of the MGBA regulatory network. Furthermore, screening gut-derived peripheral biomarkers will promote the development of individualized and precise intervention strategies for ASD.

## Figures and Tables

**Table 1:** Differences in gut microbiota between children with ASD and healthy controls detected by different techniques.

Method	ASD (Male, *n* (%))	TD (Male, *n* (%))	Region	Increased Taxa	Decreased Taxa	References
16S rRNA	143 (90.9%)	143 (88.8%)	China	*Dialister*, *Escherichia-Shigella*, *Bifidobacterium*	*Bacteroidetes Prevotella 9*, *Megamonas*, *Ruminococcus 2 Bacteroides* spp. *Prevotella* spp., *Phascolarctobacterium* spp.	[11]
16S rRNA	197 (79.1%)	52 (51.5%)	Korea	*Intestinibacter bartlettii*, *Megamonas rupellensis group*, *Coprococcus comes* group, *Odoribacter splanchnicus*	*Clostridium symbiosum*	[12]
16S rRNA	59 (76.6%)	39 (78%)	China	*Lachnospiraceae*, *Clostridiales*, *Erysipelotrichaceae*, *Dorea*, *Collinsella*, *Lachnoclostridium*	*Bacteroides*, *Faecalibacterium*, *Parasutterella*, *Paraprevotella*	[13]
16S rRNA	677 (70.7%)	122 (75.7%)	China	*Faecalibacterium*, *Blautia*, *Eubacterium_eligens_*group, *Parasutterella*, *Lachnospiraceae_NK4A136_*group, *Veillonella*	*Prevotella 9*, *Agathobacter*	[14]
16S rRNA	60 (\)	57 (\)	USA, China	*Bifidobacterium Collinsella*	*Bacteroidota phylum*, *Prevotellaceae*	[15]
16S rRNA	22 (95.7%)	15 (71.4%)	USA	\	*Faecalibacterium prausnitzii*, *Haemophilus parainfluenzae*, *Prevotella*	[16]
Shotgun metagenomic sequencing	36 (83.7%)	17 (54.8%)	China	*Eggerthella lenta*, *Clostridium botulinum*, *Klebsiella pneumoniae*, *Clostridium cellulolyticum*	*Bacteroides vulgatus*, *Campylobacter jejuni*, *Proteus mirabilis*	[17]
Shotgun metagenomic sequencing	32 (82.1%)	33 (82.5%)	China	*Veillonella parvula*, *Lactobacillus rhamnosus*	*Bifidobacterium longum*, *Prevotella copri*	[18]
Shotgun metagenomic sequencing	36 (\)	21 (\)	Russia, Europe	\	*Barnesiella, Parabacteroides*; *Alistipes putredinis*, *Bacteroides caccae*, *Bacteroides intestinihominis*, *Eubacterium rectale*, *Parabacteroides distasonis*, *Ruminococcus lactaris*	[19]
Meta-analysis	1256 (\)	1042 (\)	Multi-country	*Parabacteroides*, *Anaerostipes*, *Faecalibacterium*, *Clostridium*, *Dorea*, *Phascolarctobacterium*, *Lachnoclostridium*, *Catenibacterium*, *Collinsella*	*Barnesiella*, *Odoribacter*, *Paraprevotella*, *Blautia*, *Turicibacter*, *Lachnospira*, *Pseudomonas*, *Parasutterella*, *Haemophilus, Bifidobacterium*	[20]
RT-PCR	33 (\)	16 (\)	Poland	\	*Bifidobacterium* spp.	[21]

**Note:** ASD: autism spectrum disorder. TD: typically developing. \: the corresponding category was not reported. Italicized text denotes Latin taxonomic names of bacteria.

**Table 2:** Physiological functions of SCFAs.

SCFAs	Primary Receptor	Physiological Functions	References
AA	FFAR2	Energy Supply: Enhances oxidative capacity, fatty acid production, and inhibits lipolysis	[42,43,44]
		Appetite Regulation: Reduces appetite and suppresses weight gain	[42]
		Immune Regulation: Attenuates inflammation and maintains intestinal epithelial homeostasis	[44,45]
PPA	FFAR3	Mitochondrial Dysfunction: Inhibits key mitochondrial enzymes and disrupts energy metabolism	[46]
		Immune Impairment: Induces neuroinflammation and microglial activation	[47]
BTA	GPR109A	Immune Regulation: Maintains intestinal epithelial homeostasis and reduces production of neuro-inflammatory factors	[48]
		Neuroprotection: Enhances synaptic plasticity	[48,49]

**Note:** AA: Acetic acid; PPA: propionic acid; BTA: butyric acid; FFAR2: free fatty acid receptor 2; FFAR3: free fatty acid receptor 3; GPR109A: G protein-coupled receptor 109A.

## Data Availability

No new data were created or analyzed in this study. Data sharing is not applicable to this article.
